# Envisaging an Effective Global Long-Term Agrobiodiversity Conservation System That Promotes and Facilitates Use

**DOI:** 10.3390/plants10122764

**Published:** 2021-12-14

**Authors:** Charlotte Lusty, Ruaraidh Sackville Hamilton, Luigi Guarino, Chris Richards, Nelissa Jamora, Geoffrey Hawtin

**Affiliations:** 1 Global Crop Diversity Trust, Platz der Vereinten Nationen 7, 53113 Bonn, Germany; ruaraidh.sh@gmail.com (R.S.H.); luigi.guarino@croptrust.org (L.G.); nelissa.jamora@croptrust.org (N.J.); 2 USDA National Laboratory for Genetic Resources Preservation, 1111 South Mason Street, Colorado State University Campus, Fort Collins, CO 80521, USA; chris.richards@ars.usda.gov; 3 Alliance of Bioversity International and the International Center for Tropical Agriculture, Via di San Domenico, 1, 00153 Rome, Italy; geoffhawtin@hotmail.com

**Keywords:** genebanks, CGIAR, plant genetic resources, conservation, breeding, genomic research

## Abstract

Genebanks were established out of a recognised need not just to provide genetic variation to support breeding objectives but to prevent crop diversity from being lost entirely for future users. Such conservation objectives may have led, over the past few decades, to a gradually diminishing connection between genebanks and current users of diversity. While there continues to be large-scale distribution of germplasm from genebanks to recipients worldwide, relatively little is known or published about the detailed trends in the demand for genebank materials. Meanwhile, the rapid expansion of the applications and uses of modern genomic technologies and approaches is, undoubtedly, having a transformational impact on breeding, research and the demand for certain genetic resources and associated data. These trends will require genebanks to be responsive and to adapt. They also provide important opportunities for genebanks to reorganize and become more efficient individually and as a community. Ultimately, future challenges and opportunities are likely to drive more demand for genetic diversity and provide an important basis for genebanks to gear up.

## 1. Introduction

When Nikolai Vavilov began to collect seeds from around the world in the 1920s and 1930s, he was a pioneer in what has now come to be known as genebanking. While he and most other early crop plant collectors primarily sought landraces and traditional varieties to use as a source of novel traits in their breeding programmes, as they continued to travel and collect, it became increasingly apparent to them that many landraces were disappearing from farmers’ fields [1]. According to Mooney [2], on a collecting expedition to Turkey in the 1940s, the plant collector Jack Harlan “encountered virtually thousands of flax varieties. When he returned 20 years later only one variety remained—and this was imported from Argentina”.

Genetic erosion was especially rapid following the Green Revolution of the 1960s and 1970s, when scientists from the International Rice Research Institute (IRRI) and the International Centre for the improvement of Maize and Wheat (CIMMYT) bred high yielding varieties of rice and wheat, which were then widely disseminated [3]. While contributing enormously to easing the food shortages of the time, the rapid spread of these varieties resulted in the replacement of many indigenous landraces, a process that continues today. Observing this led many early plant collectors and breeders to recognize the importance of creating crop collections not only as a ready source of new alleles for genetic improvement, but also to conserve crop variation for the future, as an insurance against its loss in the wild and in farmers’ fields. To meet this dual need, specialized facilities for seed processing, storage and distribution were created, known as genebanks.

As the Green Revolution was taking off, new international agricultural research centres, modelled on IRRI and CIMMYT, were being established around the world and were funded collectively by a consortium of donors called the Consultative Group on International Agricultural Research (CGIAR). Those centres that were concerned with the genetic improvement of crops began to develop germplasm collections, focussed initially on providing genetic variation for the immediate needs of their breeding programmes but increasingly, over time, had a long-term conservation objective as well.

The chickpea, lentil and faba bean collections of the International Centre for Agricultural Research in the Dry Areas (ICARDA) provide one such example. They were originally assembled in Lebanon and Syria by the food legume breeders themselves to ensure that sufficient genetic variation was available to initiate their crop improvement programmes. Large collections were built up both through extensive field collecting in West Asia and North Africa, starting in 1975, and through acquisition, especially from the collections that had been put together by the USDA/USAID-funded Regional Pulse Improvement Project in India and Iran in the 1960s. Over time, as the breeders identified ever more accessions likely to provide alleles for traits of interest to their breeding objectives, so they increasingly came to spend more of their time working with this material and less time looking for additional genetic variation. However, ICARDA recognized that the collections that had been built up were not only of immediate use but had a long-term value for both the Centre’s own breeding programmes as well as those of their partners. Thus, in 1983, ICARDA established a Genetic Resources Unit (GRU) to develop and run a genebank [4]. At this point, objectives began to diverge somewhat, with conservation becoming the main priority of the GRU and developing genetically improved varieties the priority of the breeding programmes. While the GRU still aimed to serve the needs of the genetic improvement programmes, in practice, the breeders made fewer forays into the collections as they came to concentrate more on their own ‘elite’ genepools. The GRU thus took on a life of its own, with a leadership separate from that of the breeding programmes.

A somewhat similar situation occurred at the International Rice Research Institute (IRRI) in the Philippines, where the international rice collection was first assembled in the early 1960s to support the Centre’s breeding work. However, in the late 1960s, the geneticist T.T. Chang (one of the members of the team that created the semi-dwarf variety IR8, the key rice variety of the Green Revolution) had a disagreement with the breeders over what to do with material from the breeding programme that was not of immediate interest. As a result, he established the International Rice Germplasm Centre, now called the T.T. Chang Genetic Resources Centre (GRC), as a separate entity within IRRI to conserve germplasm samples that could be of longer-term value. At the same time, there was growing concern in the Philippines and elsewhere over the replacement of local rice landraces and farmers’ varieties by IR8. Recognizing this, in the 1970s, T.T. Chang began a large programme of collecting from farmers, with the primary aim of protecting against genetic erosion. The fact that the target was to conserve rather than immediately support genetic improvement contributed to a growing divide between the breeding programme and the GRC.

Since then, the breeder-genebank relationship at IRRI, as in many other CGIAR Centres, has fluctuated widely and for a variety of reasons. In the 1990s, fears over the potential impact of the Convention on Biological Diversity (CBD) resulted in reduced internal distribution of material from the genebank to the breeders as concerns grew that IRRI’s breeding lines might become subject to CBD restrictions. Later, with the adoption of the Nagoya Protocol in 2010, uncertainty over how to implement its rules on access and benefit sharing (ABS) resulted in the breeders being reluctant to deposit any of their breeding lines in the GRC. In addition, independently of political concerns, some breeders are averse to ‘polluting’ their breeding populations with ‘inferior’ germplasm from the genebank due to linkage drag.

On the positive side, a very large proportion of the IRRI collection has been screened by breeders, geneticists, and physiologists for a wide range of characteristics. In many cases, this has resulted in the identification of an allele or alleles that have enabled breeders to overcome particular bottlenecks in meeting their specific genetic improvement objectives; notable examples include “scuba rice” containing a submergence tolerance gene from a traditional Indian variety, and numerous disease resistance genes identified in and transferred from the wild relatives of rice [5,6].

The slightly ambivalent relationship between breeders and genebanks is not exclusive to the CGIAR but is widely observed in countries around the world. Greater global recognition of the social and cultural relevance of landraces and farmers varieties has given germplasm collections an importance beyond just serving as a resource for plant genetic improvement. This is particularly true at the local and national level, where the conservation of indigenous germplasm has acquired a political dimension that is reflected in the often-acrimonious debates on access and benefit sharing in various international fora, including the Nagoya Protocol and the International Treaty on Plant Genetic Resources for Food and Agriculture (Plant Treaty) [7]. On the one hand, the heightened interest in plant genetic resources has served to underline the importance of conservation, but on the other hand, a growing recognition of its potential value has resulted in greater restrictions on the ability of breeders to access material held within the collections [8].

Given this backdrop, we explore in this article how advances in genomics will further affect the relationship between the genebank and breeders, changing the way genebanks may be used in the future and creating opportunities for collections to be curated differently to maximize both their current usefulness and their efficiency in the long term. Firstly, however, we briefly describe what we know about the use of CGIAR genebanks.

## 2. Current Use of Material in CGIAR Genebanks

Systematic documentation of the distribution of plant genetic resources for food and agriculture (PGRFA) began in 2007 when the Governing Body of the Plant Treaty determined that providers of PGRFA must report to the Governing Body on the material they provide under the multilateral system of access and benefit-sharing (MLS) [9]. However, details of these reports are confidential, and only aggregated statistics are publicly available [10]. Indeed, general information on germplasm distributions from genebanks is restricted to a basic set of parameters, such as number of requests, number of accessions and samples distributed, and countries receiving germplasm. Deficiencies in data on germplasm flows for informing policy-relevant analysis and guidance was recently highlighted by Mekonnen and Spielman (2021). Private-sector recipients frequently do not want the details of their germplasm requests made known. Furthermore, the Standard Material Transfer Agreement (SMTA), which is issued as part of the transaction between providers and recipients upon the transfer of materials that are included in the MLS, expressly requires the provider to undertake that the material is provided “without the need to track individual accessions” [11].

Such principles and practices aiming to facilitate international germplasm movement and exchange have worked somewhat as a disincentive for genebanks to document and analyse information about user demand and potential future needs for genebank materials, resulting in much less being known about the current or potential deployment of diversity from genebanks than might be expected from a typical service provider.

A voluntary mechanism for uniquely identifying individual samples of PGRFA, which incorporates the possibility to track the movement and use of individual genebank materials and their derivatives, has recently been established by the Plant Treaty Secretariat with unique digital object identifiers (DOIs). At the time of writing, DOIs have been registered for 1,181,758 samples of PGRFA [12] by 2820 registrants [13], including all CGIAR genebanks and some national genebanks, but DOIs have yet to be adopted on a wide scale by genebanks or by CGIAR breeders and researchers and other users of genebanks. Once fully adopted, DOIs have the potential, at least, to allow the tracing of the use of specific accessions in published research and in germplasm exchanges and released materials [14].

The Global Crop Diversity Trust (Crop Trust) also records the numbers of samples and accessions distributed from international genebanks receiving long-term funding (including nine CGIAR genebanks). Although these efforts do not provide the kind of market intelligence that may help genebank managers and staff cater to trends or manage the collections more rationally, some patterns may be discerned.

In 2020, CGIAR breeding programs and genebanks accounted for 89% of the germplasm distributed in the MLS [15]. During the 8-year period between 2012 and 2019, a total of 853,808 germplasm samples were distributed from CGIAR genebanks, of which just over half were requested by CGIAR scientists and 47% were shipped outside CGIAR to an average of nearly 2000 requesters annually (Table 1).

The only genebank system to distribute more is the US Department of Agriculture National Plant Germplasm System, which distributes about 250,000 samples yearly, of which 25% are distributed internationally [16]. Trends in germplasm distribution from CGIAR over the past four decades have been volatile, though with a gradual upward trend [15]. Germplasm-related projects (e.g., large-sale sequencing/genotyping of rice, wheat and maize) are responsible for some of the recent peaks in demand.

**Figure 1:**
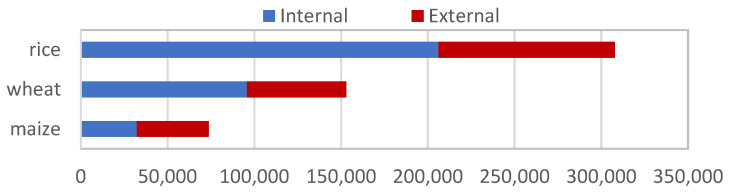
Distribution of germplasm samples of rice, wheat and maize shipped from CGIAR genebanks between 1980 and 2019 to users who are internal or external to CGIAR.

Rice is, by some margin, the most distributed of the 29 crop species (which include banana and plantain, Bambara groundnut, barley, beans, cassava, chickpea, cowpea, faba bean, temperate and tropical forages, fruit and multi-purpose trees, grasspea, groundnut, lentil, maize, various underutilized legumes, pea, pearl millet, pigeon pea, potato, rice, small millets, sorghum, soybean, sweetpotato, wheat, yam, and Andean roots and tubers) conserved by CGIAR genebanks, accounting for 36% of total CGIAR germplasm distributions between 2012 and 2019 (Figure 1).

Rice and wheat together made up more than half (54%) the germplasm distributions from CGIAR genebanks between 2012 and 2019, with the majority (65.5%) of rice and wheat samples going to CGIAR breeders and researchers (Table 1). Thus, CGIAR wheat and rice breeding and research accounted for more than one-third (35%) of the overall germplasm distribution from CGIAR genebanks. Future research efforts by CGIAR on these two crops will, no doubt, continue to have a significant influence on CGIAR genebank use.

However, it is possible to imagine that future use of CGIAR genebanks may change, given the growing range of crops of interest in agricultural research and development. The data on the distribution of germplasm of CGIAR’s mandate crops other than wheat and rice hint at the potential for such a change. Over the same 8-year period, the germplasm of mandate crops other than rice and wheat have been predominantly distributed to external users (62%) rather than to CGIAR breeders (Table 1, Figure 1).

**Figure 2:**
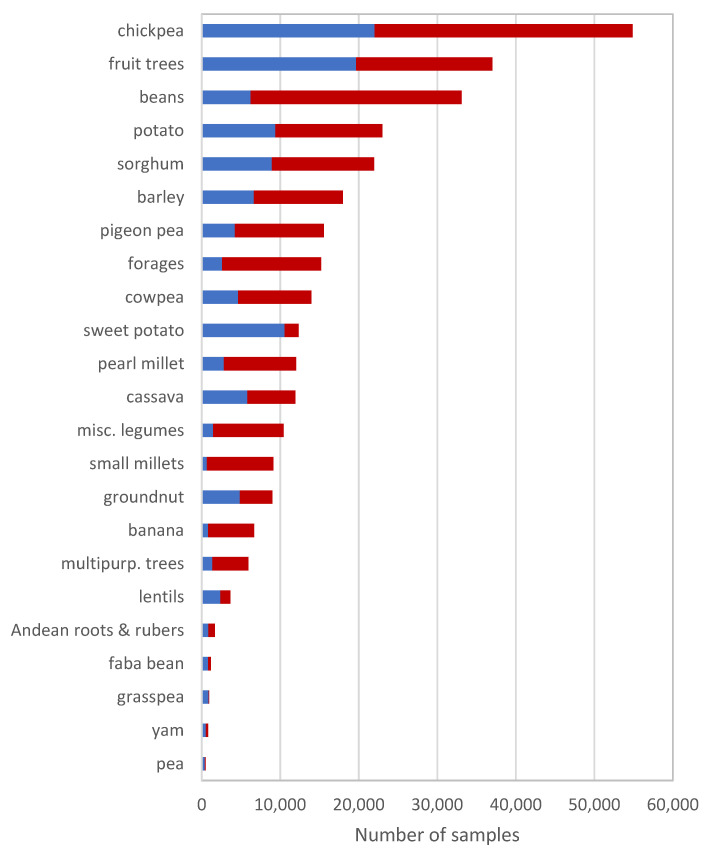
Distribution of germplasm samples of crops (exc. rice, wheat and maize) shipped from CGIAR genebanks between 1980 and 2019 to users who are internal or external to CGIAR.

Between 2017 and 2019 (when data are available), the external demand came mainly from the public sector: 81% was from national agriculture research institutes, universities and advanced research institutes, 12% were farmers, NGOs and individuals and 7% were commercial sector users (Figure 2).

**Figure 3:**
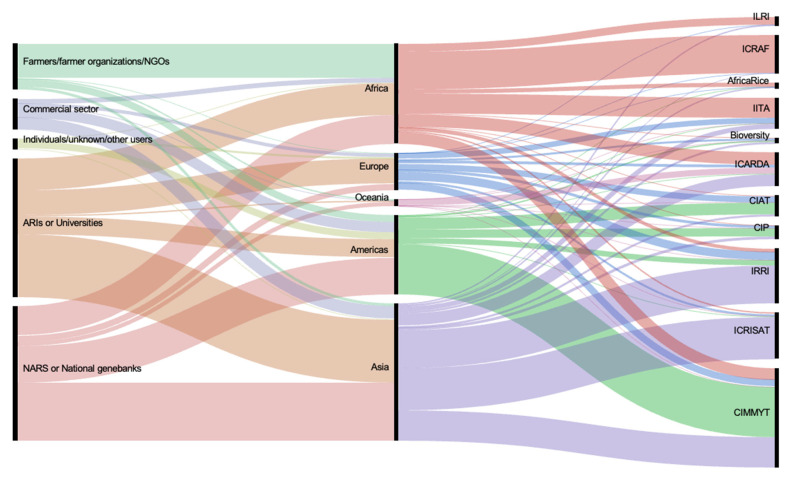
Numbers of germplasm samples distributed by CGIAR genebanks to external users according to geographical region and recipient type between 2017 and 2019.

**Figure 4:**
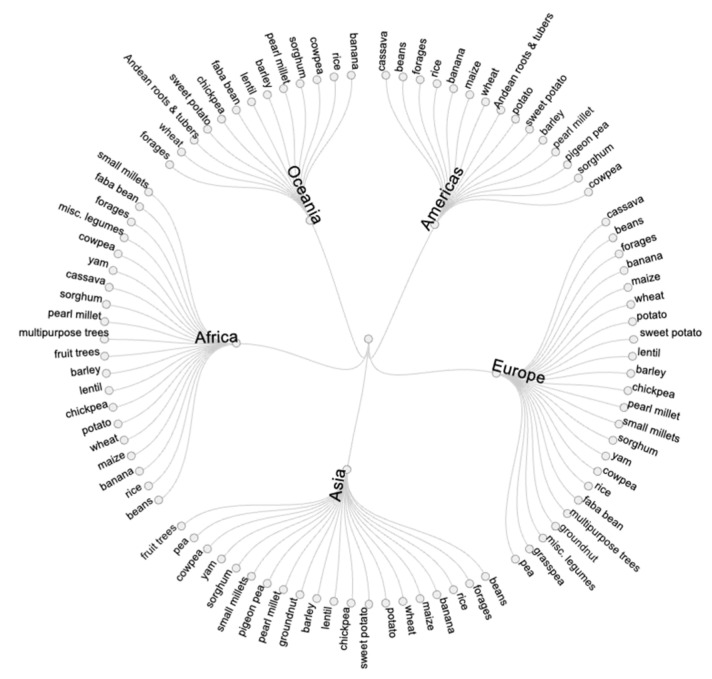
Crop species distributed by CGIAR genebanks to different geographical regions between 2017 and 2019.

Germplasm samples were shipped to every region and sub-region of the world in response to requests for a diverse range of crop species (e.g., 20 species were shipped to both Africa and Asia). Asia received the most germplasm samples (42%), followed by Africa (23%) and the Americas (19%) (Figure 3 and Figure 4). All regions show similar proportions of germplasm going to different user categories, although most of the samples requested by farmers and NGOs were shipped to Africa.

**Figure 5:**
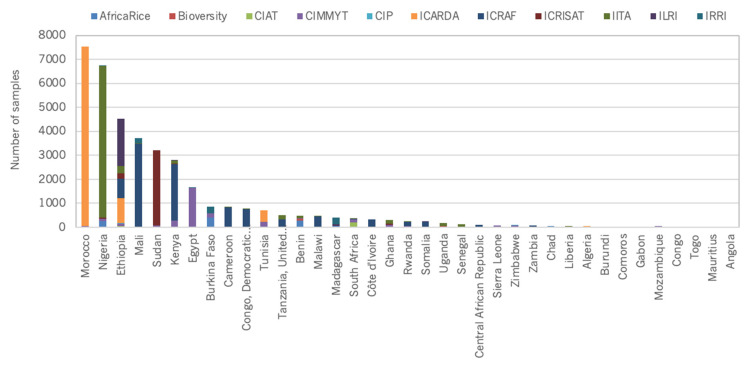
Number of germplasm samples distributed by CGIAR genebanks to external users in Sub-Saharan Africa.

**Figure 6:**
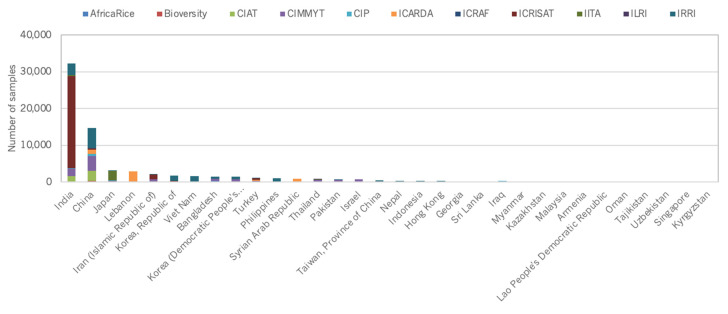
Number of germplasm samples distributed by CGIAR genebanks to external users in Asia.

**Figure 7:**
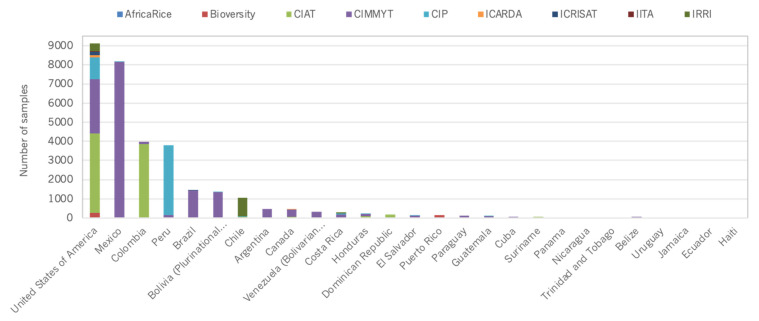
Number of germplasm samples distributed by CGIAR genebanks to external users in the Americas.

**Figure 8:**
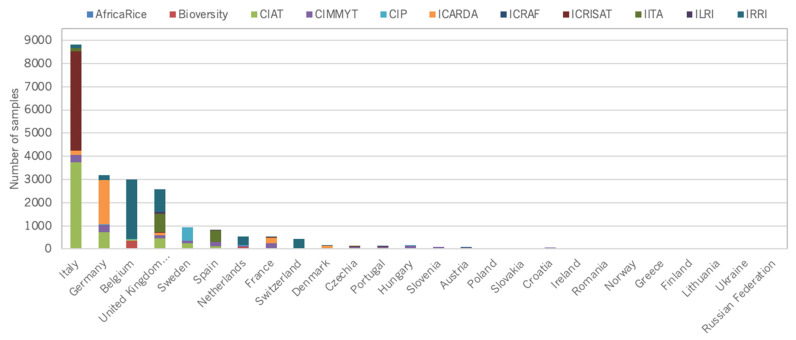
Number of germplasm samples distributed by CGIAR genebanks to external users in Europe.

At a country level, distribution figures are skewed towards countries that host CGIAR genebanks (Figure 5, Figure 6, Figure 7 and Figure 8).

In Africa, the top three recipient countries are Morocco (now hosting ICARDA), Nigeria (IITA) and Ethiopia (ILRI), and, combined, they receive more germplasm than the rest of the continent put together. Are other African countries accessing crop genetic resources from their national genebanks or genebanks other than CGIAR? The Tropical Agricultural Research and Higher Education Center (CATIE), the Centre for Pacific Crops and Trees (CePaCT), International Center for Biosaline Agriculture (ICBA) and the World Vegetable Centre have international genebanks and also provide germplasm globally, but mostly of different crop species than CGIAR. Such patterns pose many questions about germplasm distribution and demand that, for now, remain unanswered.

Given the unpredictability of future challenges and opportunities, the multitude of ways agriculture producers and consumers may respond to them, and the increasing capacity to generate knowledge to facilitate the use of genetic diversity, these patterns could suggest that there is a significant latent demand in many middle- and low-income countries for germplasm and services from CGIAR and possibly other genebanks. Without gathering more detailed data from users or potential users concerning the deployment and need of germplasm from genebanks, and analysing trends as a routine, it is difficult to improve our understanding of user demand or potential demand and respond appropriately to it. However, there are major advances in science that are impacting and will continue to impact the use of genebanks, and these, too, should have a major influence on how germplasm samples are delivered and conserved. We will now turn the discussion to these points.

## 3. Advances in Genomics and Their Influence on Breeding and the Role and Structure of Genebanks

### 3.1. Advances in Breeding

Modern breeders typically work with a limited diversity of painstakingly chosen potential parents, enabling reliable progress that does not risk breaking up the superior combinations of genes in elite breeding material. Crosses between more genetically distant parents bring the potential for much greater stepwise progress, and become essential when breeding objectives change (such as when a new disease appears) or when an existing breeding programme stagnates for lack of diversity to work with (The amount of additive genetic variation for a trait under selection in a breeding population ($\sigma_{a}$) is one of the determinants of the annual rate of genetic gain $G=\left(\sigma_{a}ir\right)/L$, where *i* = selection intensity, *r* = selection accuracy and *L* = number of years per cycle [17]). However, such “wide crosses” bring the risk (or even the near certainty in crops such as maize or in interspecific crosses between crops and their wild relatives) of breaking up desirable combinations of genes [18].

Fortunately, advances in genomics are creating new opportunities for exploring and utilizing crop diversity [19,20,21,22]. Breeders can now choose parents and select progeny based directly on genotype rather than phenotype, which can be much faster and cheaper. Selections based only on phenotype are often challenging because of the low heritability and polygenic nature of the desired traits, their high dependence on the environment in which they are assessed, and the genetic background of the genes involved. In contrast, for single-gene traits for which there are good markers (Ideally markers are within the gene controlling the trait, but they do not have to be. Good markers are often close to (and therefore genetically linked with), but not part of, the gene controlling the trait. The further the marker is from the gene, the less tightly it is linked, and therefore the more dependent on the specific materials being bred.) for the desired functional genetic variants, the required genotypes can be selected with high certainty at the seedling stage.

In addition, through gene editing it is now possible to add, delete or change a single gene in a genome. Where a causal relationship has been established between a single gene and a desired trait, this makes it possible for breeders to add a high-value gene (e.g., the sub1 gene responsible for “scuba” rice, see above) into a high-value genome in a single step, enabling large improvements without the risk of breaking up desirable gene combinations. In conjunction with synthetic biology, it will eventually become possible for breeders to even edit the gene without accessing physical material in genebanks. However, gene editing is an effective breeding tool only after research to determine the sequence and function of the “best” functional genetic variant for any given objective. This research will rely on continued access to physical genetic resources for the foreseeable future. Additionally, gene editing typically addresses only one gene at a time or about 0.002% of the genes in the genome (although techniques are being introduced to edit several genes simultaneously [23]), but it is much faster and more precise than conventional backcrossing. For traits with complex or uncertain genetic control, genomic selection may be used [24]. The prerequisites in this case are an effective, intelligent algorithm for the selection of variants across the whole genome and a good training population for the algorithm to learn from. Genomic prediction also requires high-throughput phenotyping to arrive at, and to validate, the algorithms [25].

Advances thus continue to bring gains in efficiency and effectiveness to the slow-but-sure approach of modern breeding, but there remains a glaring need to explore the much greater potential of recombining widely different genomes—an area where genebanks can uniquely contribute. The client base for genebank material is increasingly shifting from breeders towards upstream researchers. There is a rapidly emerging client base for “digital genebanks”, i.e., for comprehensive online searchable repositories of information on genetic resources, as users increasingly require access to digital information associated with accessions. It is clear that genebanks will need to evolve, not only to improve how they work and catch up with the advanced state of breeding (particularly for the most advanced crops, although under-utilized and less intensively bred crops will follow), but also to accommodate a changing role [26,27,28,29].

### 3.2. Advances in the Role of Genebanks

Given the advances in breeding and genomics described above, we can envisage a not-too-distant time when every gene or haplotype (including coding, non-coding and regulatory regions) within the crop genepool being conserved will be catalogued and searchable, along with every existing potentially functional variant of each of these. The development of a comprehensive catalogue of the functionally significant genetic variants of each accession can thus become a feasible target for the ideal genebank of the future. Many of those variants will have their phenotypic effect either predicted or empirically demonstrated in at least one environment, genetic background and epigenetic status, or at least imputed from their homology to other known sequences. Whole genome sequences help reveal functional variants, including structural variations such as inversions and deletions that are hard to identify and map using conventional methods but may have significant impact on phenotypes. Pangenomics analyses enable the discovery of such variations that cannot be seen with genotyping. Even with as many as a million genomes per crop for 20 crops, with around 25,000–75,000 genes per crop genome, the data in the catalogue might require only about 20 terabytes of storage capacity (Very approximately and subject to revision: a million genomes per crop for 20 crops gives 20 million genomes. Multiplying 20 million genomes by 50,000 genes per genome gives a trillion records. Each record would be a pointer to an entry in a dictionary of gene variants: at, say, 20 bytes per record, that is 20 terabytes. The dictionary itself would be a fraction of that size at about 5 gigabytes (20 crops * 50,000 genes * approximately 5000 bytes per gene based on a full sequence for the most common variant and differences for the other variants). This is tiny relative to modern “big data” applications and readily tractable. It would be a game-changing contrast to relying solely on the never-ending treadmill of phenotyping: a digital genebank that provides material and information that meets users’ needs with a precision that is currently unachievable.

Given the rate of progress to date, including automated algorithms for genome annotation, it should be possible to build an initial, reasonably comprehensive, multi-crop catalogue of functional genetic variants within 20 years. However, the catalogue would need to be progressively refined continuously after that.

In the meantime, to explore diversity and to develop the catalogue, a range of options needs to be built up to stratify collections for easier research and use. Many genebanks have already identified traditional core or mini-core subsets intended simply to make the task of phenotyping large collections more manageable [30]. Alternatively, accessions have been selected based on specific user-defined criteria (usually combinations of passport, phenotypic and genetic data), including using machine learning software such as the Focused Identification of Germplasm Strategy (FIGS) developed by ICARDA to create subsets that are more likely to contain adaptive traits that users want [31,32]. One of the reasons for higher distribution figures from genebanks for some crops, such as rice from IRRI, over the past decade has been the increased demand for subsets of accessions that have been sequenced [33,34,35,36,37]. The reason is that this enables users to conduct their own genome-wide association studies, which is an increasingly important first step in understanding the genetic control of a trait: a single sequenced subset can be used to support gene discovery for multiple traits. Hence, a short-term objective for genebanks should be to replicate this for all crops by sequencing the genomes of well-chosen core collections of all their crops.

In addition, genebanks should be invested in becoming more proactive in designing and creating novel genetic resources in support of breeders and researchers. Importantly, they must complement rather than duplicate breeders’ own trait-discovery or “pre-breeding” work, and hence must undertake such efforts in consultation and collaboration with breeders. Breeders’ pre-breeding initiatives are typically trait-specific, focusing on introgressing high-value traits from “undesirable” genomes into elite breeding lines. Genebanks may play a complementary role by “pyramiding” multiple known high-value traits into easily useable material [38]. They could also take a more exploratory or trait-agnostic approach, combining divergent genomes that have never previously been crossed with the aim of exposing large amounts of novel phenotypic diversity by creating radically different genomes, supporting rapid response to change. A range of possible crossing designs already exist, such as MAGIC (Multi-parent Advanced Generation Inter-Cross) and NAM (Nested Association Mapping); their exploratory value can be maximised by using genomic information to select the parents. Genebanks have a particularly complementary role to breeders in exploring the variation available in crop wild relatives by crossing them with elite material to tease out hidden characteristics and developing combinations that may eventually be more attractive to the breeder to work with [39].

For the purposes of gene discovery, it is important to phenotype the exact same genome (i.e., the precise individual) that has been sequenced or genotyped. This may require the sequenced genome to be managed, conserved and distributed separately from the accession from which the genome was taken. However, if many accessions are conserved in their original form and also in the form of pure lines, the size of the collection would at least double. Clearly management decisions will need to take this into account and genebanks will need to adapt to conserve such genetic stocks on a short-term basis and provide them to breeders and researchers.

### 3.3. Advances in the Structure of Genebanks

The ability to ensure delivery of materials more precisely corresponding to the demand of users will not only speed up crop improvement but vastly increase the return on investment in genebanks, and it may even lead to a change in genebank funding models. Today, contrary to normal practice for other services, whereby users pay for the services provided, genebanks are effectively paid to provide genetic resources to users by governments and donors. The justification for such public spending is compelling: users need access to genebanks to broaden the diversity of materials they use for agricultural development to everyone’s benefit, but, as the genebank cannot know which accessions will actually help any given user, users will understandably not pay for such services. Once genebanks start delivering well-targeted materials that meet users’ needs, however, the more usual “user pays” funding model may work for genebanks as well, subject to the provisions of the Plant Treaty.

If users were to pay for more precisely attuned services, genebanks would need to learn how to place a value on the resources they conserve and provide. Resource economists have established ways of conceptualizing different categories of value for economic research: use, non-use and option values [40]. In the future, new tools could help to quantify the value of germplasm appropriately. Advanced algorithms, based on genome-wide selection, may be used to explore the likely consequences of combining different genomes. The result would be a purpose-specific “current value”, or “use value”, for each accession, i.e., the extent to which that accession could enable a breeder or researcher to meet their known current needs. These values would be highly dynamic, increasing as accessions are found to contain genes needed by the breeders, and decreasing as those genes enter the breeders’ own genepools. They would be used to select the most appropriate materials for specific current users. They could also be used more proactively to guide the creation and management of a large, dynamic set of user-oriented accessions, pre-bred by the genebanks (or others) and designed to meet current needs of researchers and breeders as effectively as possible.

However, such a focus on current value must not detract from the role of genebanks in long-term sustainability. Other measures of accession value must also be introduced to ensure an effective long-term agrobiodiversity conservation system. Unique diversity that differs genotypically from varieties in current use will have an “option value”: even if this diversity has no value for today’s food production, conserving it keeps open options for responding to future challenges as they emerge. The option value of an accession will be a function of the number of functional genetic variants (including epigenetic factors, structural variants and transposable elements) that are present in the accession but that are either not known or at risk of extinction outside the genebank. Such materials could include originally collected materials, heterogeneous accessions and populations. These accessions need to be conserved in a way that efficiently keeps their unique genes available for future use without needing to invest in their current use.

In addition, objects whose very existence is prized have a “non-use value”. This concept applies, for example, to “heirloom varieties” that may be considered part of the heritage of a particular country or culture or community and may be at risk from changing conditions, practices and priorities if not conserved in a genebank. This should not be taken to imply that heirloom varieties do not have a use value. On the contrary, their use within certain cultures may be vital for those cultures. It just means they have a value beyond their use value, as implied by the very term “heirloom”, one of the central underlying themes of non-use value. The importance of some of these types of material will clearly reside in the variety as a whole; that is, in the entire genome, rather than in specific rare genes or gene combinations.

It is important to recognize that these different values are independent concepts and are not mutually exclusive. A heritage variety may contain functional variants that breeders do not have in their collections but that would help meet their objectives and may also contain other unique functional variants with unknown value. An accession of such a variety would have high use value, high non-use value, and high option value. A well-researched and used accession that has functional variants that are already well represented in other accessions may have much less option value and, therefore, be a much lower priority to conserve long term. Purified lines are a clear example.

Beyond uncovering the genetic mechanisms underlying agronomic traits, genomic data can provide detailed analysis of the population genetic history shaping diversity in situ and the mode and tempo of selection during domestication of crop plants [41,42], as well as the long-term effects of keeping genetic diversity ex situ in a genebank. Most accessions in crop collections do not represent uniform sets of genotypes, but rather heterogeneous populations of genotypes, reflecting the mutational and migration effects that are captured at the moment at which the sample is collected. Accessions of crops’ wild relatives have inbreeding and levels of differentiation that reflect the sampling effects and logistical constraints of the collector, as well as the inherent breeding system, life history, and ecogeographical range of the species [43]. Any effort to estimate or put a value on this diversity, therefore, must take a population genetic approach to sampling and prioritization.

Curation for long-term conservation and for current use will diverge: the original, heterogeneous accessions may be a cost-effective way of conserving genes and populations long term but may have less value for current use, while the reverse is true for sequenced, purified lines. The future genebank system may be viewed as structured collections with varying levels of intra-accession diversity, different conservation objectives, and varied precision in characterization data. Wild and landrace accessions may be conserved to represent diverse populations with wide-ranging characteristics. Population genetic approaches will be best used to evaluate the diversity of these accessions. Improved accessions and genetic stocks will have increased uniformity with increasingly precise characterization data. The different levels of diversity complement each other. The more purified accessions can serve as a starting point to dissect the genetic architecture of agronomic traits and query the more diverse accessions to find useful allelic variants at key loci [44].

Parameters revealing diversity and differentiation, relatedness and admixture within the collection, and analyses that seek to understand the population genomics of domestication history will be of critical interest. In addition, rounds of regeneration subject the diversity (of individual genotypes or individual haplotypes) to sampling variation, resulting in genetic change in accessions that are not initially uniform. Because this process is driven by sampling (the larger the sample or effective population size, the lower the drift), a genebank can try and maintain genetic integrity through large regeneration populations or through extending generation time intervals between regenerations by ensuring storage conditions are optimized for the long term [45].

Deriving the current use value of accessions is not yet achievable and will, of course, depend on having access to that digital genebank of functional variants. Even with the future digital genebank, details of how to assign a current use value to each accession will depend on various factors, including progress in the development of genetic algorithms, experience in the extent to which genomic predictions must be supported by direct phenotypic observations, and evolution of the ways in which genebanks monitor the changing needs of users. Before providing a practical way of managing germplasm collections, much further work will be needed to develop methodologies to quantify current value. What is important to recognise, however, is that once users are able to more effectively select the materials they need from genebanks, there will be a need to re-structure collections in ways to accommodate fast access to accessions and research-ready materials with a relatively high turnover compared to conventional genebank collections. A well-established collection would be expected to contain a relatively small and stable set of accessions with high option value and high non-use value. It would contain a larger and more dynamic set of accessions with high current use value, changing as users’ needs change. New accessions would only be added to the genebank’s collection where assessment of their potential value demonstrates that they add significantly to the collection’s overall value. The technology already exists to obtain a genome sequence in the field in real time [46]; this would be used to sequence a sample and determine if the sample should be added to the collection or discarded, based on its complementarity to the existing collection.

Deriving the future use value of accessions faces different challenges. Whilst not requiring phenotypic information, it does require consideration of within-accession heterogeneity. However, methods to study the genomics of variable accessions are limited. Attempting to discover the full set of functional variants within one accession by genotyping every individual is not a viable option. This will need to be taken into account in developing methods for handling within-accession heterogeneity, rare functional variants, and their contribution to the future use value of an accession.

A picture starts to emerge whereby the distinction between long-term conservation and immediate use in genebanks will become more pronounced and functional. Up until now, general practice and guidelines have divided genebanks into base collections made up of relatively small samples of the most original seeds held in long-term storage conditions (at a temperature of −18 ± 3 °C and relative humidity of 15 ± 3%) and larger, more dynamic, so-called “active” collections that hold larger samples for distribution and use under refrigeration (at 5–10 °C and relative humidity of 15 ± 3%) [28]. There are exceptions; some genebanks (e.g., CIAT) hold their collections entirely in long-term storage conditions in batches destined for different purposes (e.g., long-term storage, viability monitoring, repatriation, distribution and safety duplication). However, in most cases there is good economic sense in making the distinction between samples that can be left relatively undisturbed for long-term conservation and those for immediate use, since long-term storage at −20 °C is slightly more expensive to run and cannot be staffed in the same way as medium-term storage at 5 °C because of the working conditions. It does not necessarily follow that materials that are held in long-term storage conditions are bound to be conserved for the long term. However, in practice whatever is in long-term storage tends to be challenging to discard and, thereby, becomes a long-term obligation to conserve.

**Figure 9:**
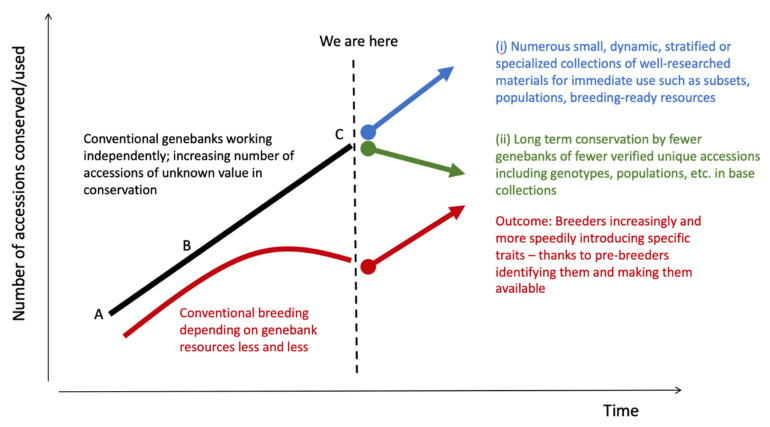
The evolution of genebanks over time—(**A**) originating as a breeding resource in the 1950–60s, (**B**) transforming into the subject of long-term conservation as well as a breeding resource, (**C**) evolving into (i) specialised, dynamic collections for breeders and other users and (ii) base collections for long-term conservation.

In future, with in depth analysis of the genetic composition of collections, samples of high option value accessions may be prioritized for long term conservation. By contrast, for immediate use, smaller, more dynamic collections of breeder-ready resources, populations, trait subsets, phenotyped core collections, purified lines and other high current value materials that are the subject of active research and phenotyping will be maintained in various derived forms (i.e., not necessarily in the form in which they were collected) (Figure 9).

Given this vision, it is not a giant leap to suggest that genebanks will have significant opportunities to concentrate conservation activities and to specialise. Specialist genebanks already exist; ICBA is developing a specialist collection for salt tolerance, and different CGIAR genebanks focus on tropical agriculture, drylands, semi-arid regions and specific crops. In the future sketched out above, a limited number of genebanks would need to focus on, and specialise in, the long-term conservation of particular crop types: orthodox seeded crops, clonal crops, fruits and vegetables, and wild species, for example. Specialisation would facilitate deep innovations in crop and germplasm management protocols to improve quality, increase reliability, enlarge capacity, and reduce costs. If there are highly repetitive tasks, there are possibilities to automate them for consistently high quality, high throughput, and low cost. Automated processes and better materials management also introduce the possibility of tighter control through remote management, allowing genebank curators to control processes no matter where they are—an advantage that has shown its relevance during pandemic lockdowns. The composition of staff would change, with new technical expertise required for machine and process maintenance, and there would also be a shift in staff balance, with a higher proportion dedicated to information processing and management.

IRRI has recently piloted the automation of rice seed sorting. In collaboration with the private sector, the genebank has developed bespoke robotic seed imaging machinery that can be trained for each individual accession to sort high quality seed for storage [47]. Some preliminary attempts have been made to automate various other operations in large genebanks, for example planting, phenotyping, harvesting, viability testing, packing, labelling, and the storage and retrieval of materials from a seed store [48]. The most widely adopted and successful advance, so far, has been the introduction of bar-coded or QR-coded labels for inventory management and for tracking samples through workflows. Generally speaking, it is considerably more challenging to automate genebanks managing multiple crops with diverse, heterogeneous accessions. Only concentration into fewer, larger collections, enabling higher throughput, will tilt the balance towards more automation being appropriate and effective.

## 4. From Vision to Reality

Comparing today’s germplasm distribution data with the vision that we have described above reveals what appears to be an abyss or, perhaps less dramatically, a mismatch between theory and reality. It is important, firstly, to note that distribution statistics will never accurately reflect the use of genebank materials and data or their impact. Distribution is merely the first step in use, not the end result. Nevertheless, a more accurate method of gathering and monitoring germplasm distribution data will be essential in informing the directions to be pursued by genebanks and the institutes and donors that support them. We need basic but detailed and consistent data on every genebank request: the type of material requested, when, by whom, for what purpose, and under what ABS conditions.

However, feedback from users about the use and performance of distributed germplasm samples would also be highly desirable, though it presents significant legal and technical obstacles. The SMTA prohibits providers from requiring such feedback from users of genebank accessions (but allows it for breeding lines that can be categorised as “PGRFA under development”). Hence, traditional attempts to promote feedback will never be particularly effective. On the other hand, the SMTA obliges users to provide such feedback through the Global Information System (GLIS). This potentially opens the door to an effective system, although the GLIS currently has only a rudimentary mechanism to receive feedback. This mechanism relies on GLIS DOIs being used to identify the material and on those DOIs being used by breeders in publications and in online datasets. The only legal and operational mechanism currently available to obtain feedback on the use and performance of distributed germplasm samples is DOIs. Everyone who believes crop diversity and the genebanks that conserve it are important should promote the use of DOIs by germplasm users.

Distribution data hints that there is a wide range of current users, but potentially many more users who may want to request a wider range of genebank materials and crops. CGIAR and other genebanks should not only be gathering and curating more and better data on existing requests, but actively scanning and assessing potential and future demand by better characterising and understanding their users, their users’ capacity and their germplasm needs. The reasons behind the apparent geographic patterns of distribution should be better understood. This means a more proactive effort to engage users, follow up after requests, analyse demand patterns and identify potential users; carrying out survey work and promotional work; and collaborating closely with activities to gather market intelligence to determine breeding priorities.

Genebanks will need to meet the needs of users both in terms of genetic resources and associated data more accurately and efficiently than they do today, including those of a burgeoning community of upstream researchers needing both material and in-depth genomic information. As well as responding to requests, genebank activities need to proactively explore hidden traits in collections and develop breeding-ready subsets and resources more closely matching analysed needs. Interacting more closely with the user community, and in particular those involved in pre-breeding, will be crucial to ensure that genebanks conserve the right genetic resources in the right way and closely match resources to the priorities. It is important to stress that these activities need to be funded and should not take the place of important ongoing conservation work.

Although only acquisition and curation have been discussed here, all other genebank processes must become more dynamic as well. Procedures for managing materials, information and processes must be streamlined to maximize efficiency, maintaining consistently high and demonstrable quality while reducing costs in a system that matches throughput capacity to demand. CGIAR recently endorsed a policy framework for the strategic curation of collections under its management, involving the establishment of different curation categories, including the option to “partially curate” or “archive” accessions, formalizing a practice that many genebanks have had in place for years that allows them to adapt the usual sequence of genebank processes for specific accessions where appropriate.

As CGIAR evolves under the current One CGIAR reform, its genebanks will continue to play a pivotal role in a global system for the conservation and use of genetic resources and have an opportunity to contribute to fulfilling the vision outlined above in a number of ways, including the following:

- Providing facilities for the effective management of long-term conservation of an increasing number of crops, and collaborating with others, including the Svalbard Global Seed Vault, in this process. Through enhanced collaboration, consolidation, and division of labour, possibly also involving the private sector, it should be possible to significantly increase the efficiency and effectiveness of long-term conservation;
- Developing novel diversity, e.g., through wide and inter-specific crossing, and creating value-added subsets of materials for breeders, e.g., for genome-wide association studies. Again, there should be scope here for enhanced partnership with private companies;
- Developing methods for assigning current and future values to accessions and for using such values for decision making with respect to curating conserved materials and promoting use, taking into account within-accession heterogeneity;
- Working with national, regional and international partners to develop a system of distribution hubs so as to more efficiently and effectively provide germplasm to those that need it around the world. This is likely to involve the maintenance of dispersed active collections linked to facilities for ensuring the health status of distributed germplasm [49];
- Large-scale sequencing of accessions of all mandated (and, in time, other) crops and making this information available in conformity with applicable ABS regulations;
- Providing input to the future development of international policies, rules and regulations regarding the conservation and use of plant genetic resources, including the equitable sharing of benefits arising from such use;
- Promoting the use of GLIS DOIs by all users and providers as the globally unique public identifier for germplasm samples;
- Providing the training needed within CGIAR and partner institutions and securing adequate financial and other resources to enable this vision of the future to become a reality.

## 5. Conclusions

This is an extraordinary time of change throughout the world. Climate change and the consequent increase in extreme weather events is already having a significant impact; biodiversity is disappearing despite massive efforts to conserve it; Covid-19 has resulted in a huge increase in human misery and slowed down large sectors of the economy; and the increasing polarization of society and political views is threatening long-established governance systems. To exacerbate things, the UN has estimated that the world’s population will grow by almost 1.9 billion people between 2021 and 2050. Plant genetic resources for food and agriculture have an important role to play in addressing many challenges by the development of higher yielding, more nutritious and resilient crops that can help increase rural incomes and avert malnutrition and social unrest, and of crops and cropping systems that require less land and fewer external inputs, or that release less greenhouse gasses.

As this article has attempted to show, future plant breeders are likely to require more, not less genetic diversity than at present, but in a different form and accompanied by larger amounts of reliable data. It is probable that demand will continue to grow for genebank materials that can be used in gene discovery and for the identification of functional variants, shifting the client base toward more upstream scientific researchers. If genebanks are to remain relevant, it will be important that they are able to adapt and cater to new demands. This has important implications for the types of material they maintain and the form in which it, and the associated data, are made available. At the same time, new conservation technologies, policies and institutional arrangements offer ways to improve the efficiency and effectiveness of conservation to the long-term benefit of all.

## Figures and Tables

**Table 1:** Germplasm distribution from CGIAR genebanks between 2012–2019, showing the proportion of rice and wheat compared to the other 27 mandate crop species that are available from CGIAR and the proportion of samples shipped outside CGIAR to users making requests.

Column1	Total	Annual Average	Rice & Wheat	% Rice & Wheat	Other Crops	% Other Crops
Total number of samples distributed	853,808	106,726	460,976		392,832	
**% of total distributed**			**54%**		**46%**	
Number of samples distributed internally within CGIAR	452,966	56,621	301,942	**66%**	151,024	**38%**
**% of total internally distributed**	**53%**		**65.5%**		**38%**	
Number of samples distributed to users outside CGIAR	400,842	50,105	159,034	**34%**	241,808	**62%**
**% of total externally distributed**	**47%**		**34.5%**		**62%**	

## Data Availability

Data on germplasm distribution from CGIAR genebanks are available at www.genebanks.org/resources/genebanks-in-numbers/distribution/ and from the genebanks online reporting tool made available upon request to nelissa.jamora@croptrust.org.
